# Supplementary material for: EnzML: multi-label prediction of enzyme classes using InterPro signatures
Source: BMC Bioinformatics. 2012 Apr 25;13:61. doi: 10.1186/1471-2105-13-61 (PMC3483700; doi:10.1186/1471-2105-13-61)
Supplement: Addtional file 5 — The Java code to format the data files, evaluate and predict. The file enzml_java_code.tar.gz contains the Java code used to format database data to ARFF and XML formats, to execute cross and train-test (jackknife) evaluations and to record evaluation results to database. More information is included in the readme.txt file and the Javadoc files. The code can be used with a MySQL database. To use a different database software, other JDBC drivers might be required. [file 1471-2105-13-61-S5.gz › java_code/utils/doc/uk/ac/ed/inf/utils/database/class-use/TableColumn.html]

Uses of Class uk.ac.ed.inf.utils.database.TableColumn


---


|  |  |  |
| --- | --- | --- |
| **Overview** \| **Package** \| **Class** \| **Use** \| **Tree** \| **Deprecated** \| **Index** \| **Help** | |  |
| PREV   NEXT | **FRAMES**    **NO FRAMES**     **All Classes** |  |


---


## **Uses of Class uk.ac.ed.inf.utils.database.TableColumn**

| Packages that use TableColumn | |
| --- | --- |
| **uk.ac.ed.inf.utils.database** |  |

Uses of TableColumn in uk.ac.ed.inf.utils.database

| Methods in uk.ac.ed.inf.utils.database that return TableColumn | |
| --- | --- |
| `static TableColumn` | `DbUtils.getIdColumn()`             get the definition for an "id" column of type auto-increment (which is primary key) |
| `static TableColumn` | `DbUtils.getTimeDownloadedColumn()` |
| `static TableColumn` | `DbUtils.getTimestampColumn()` |

| Methods in uk.ac.ed.inf.utils.database that return types with arguments of type TableColumn | |
| --- | --- |
| `java.util.Vector<TableColumn>` | `Table.getColumns()`             Get the `TableColumn` list |

| Methods in uk.ac.ed.inf.utils.database with parameters of type TableColumn | |
| --- | --- |
| `int` | `TableColumn.compareTo(TableColumn f2)`             Compares two columns by their name, returns -1 if this column comes alphabetically before f2, 0 if the two are equal and +1 if f2 comes alphabetically before this column. |
| `boolean` | `TableColumn.equals(TableColumn anotherColumn)`             Compares two columns by their name, returns true if the two columns have the same name, false otherwise. |

Constructor parameters in uk.ac.ed.inf.utils.database with type arguments of type TableColumn

`Table(java.lang.String tableName,
java.util.Vector<TableColumn> columns)`
  
          List of primary key fields (starting from 0)

---


|  |  |  |
| --- | --- | --- |
| **Overview** \| **Package** \| **Class** \| **Use** \| **Tree** \| **Deprecated** \| **Index** \| **Help** | |  |
| PREV   NEXT | **FRAMES**    **NO FRAMES**     **All Classes** |  |


---
